# Erratum to: Acaricidal effect and histological damage induced by *Bacillus thuringiensis* protein extracts on the mite *Psoroptes cuniculi*

**DOI:** 10.1186/s13071-015-1013-0

**Published:** 2015-08-18

**Authors:** Emmanuel Dunstand-Guzmán, Guadalupe Peña-Chora, Claudia Hallal-Calleros, Mario Pérez-Martínez, Víctor Manuel Hernández-Velazquez, Jorge Morales-Montor, Fernando Iván Flores-Pérez

**Affiliations:** Facultad de Ciencias Agropecuarias, Universidad Autónoma del Estado de Morelos, Av. Universidad 1001, Col. Chamilpa, Cuernavaca, 62209 Morelos Mexico; Centro de Investigaciones Biológicas, Universidad Autónoma del Estado de Morelos, Av. Universidad 1001, Col. Chamilpa, Cuernavaca, 62209 Morelos Mexico; Facultad de Medicina Veterinaria y Zootecnia, Universidad Nacional Autónoma de México, Av. Universidad 3000, Col. Copilco, Ciudad de México, 04510 Mexico; Centro de Investigación en Biotecnología, Universidad Autónoma del Estado de Morelos, Av. Universidad 1001, Col. Chamilpa, Cuernavaca, 62209 Morelos Mexico; Departamento de Inmunología, Instituto de Investigaciones Biomédicas, Universidad Nacional Autónoma de México, AP 70228, México, DF 04510 Mexico

The original version of this article [1] unfortunately contained mistakes. Panel headings in Figs. 3, 4 and 5 (Figs. 1, 2 and 3 here) were included incorrectly.

**Fig. 1:**
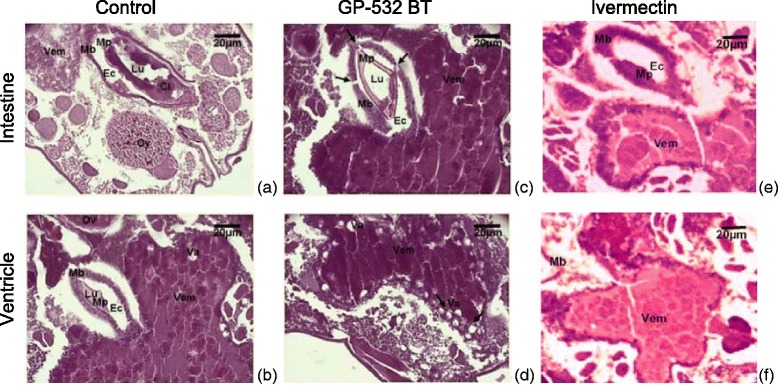
Photomicrograph of longitudinal sections in control (**a**, **b**), treated mites with the strain GP532 of *B. thuringiensis* (**c**, **d**) or with ivermectin (**e**, **f**). LU: lumen of the intestine, MP: peritrophic matrix, EC: ectoperitrophic space, MB: basement membrane, CI: intestinal content, Va: vacuole, VEM: medium ventricle, Ov: ovary. Arrows indicate alteration on the intestinal basement membrane or vacuole shaped fat deposits in ventricle, H&E- X40

**Fig. 2:**
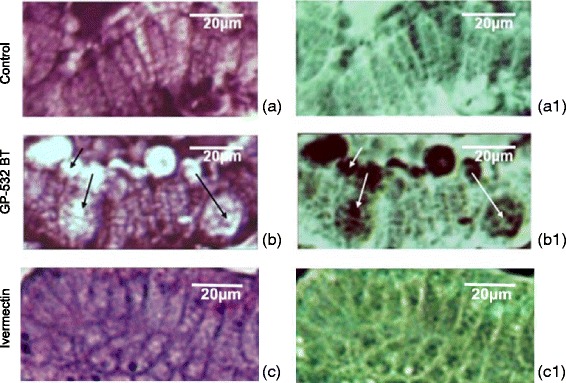
Representative image of the structure of the columnar cells in the mite *P. cuniculi* without treatment (**a**) or after treatment with the strain GP532 of *B. thuringiensis* (**b**) or ivermectin **c**, and their respective images through the relief filter (a1, b1, c1). Arrows indicate presence of dilated intercellular spaces of the columnar epithelium regarding the peritrophic matrix. Bar = 20 μm

**Fig. 3:**
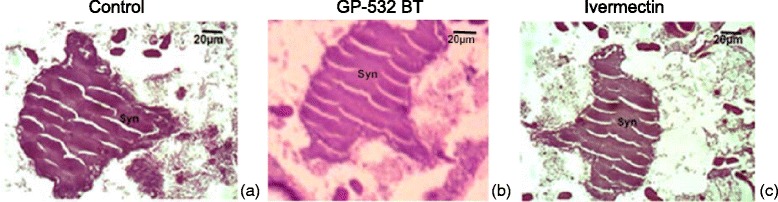
Photomicrograph of longitudinal sections in nervous system of control mites (**a**) or treated with the strain GP532 of *B. thuringiensis* (**b**) or ivermectin (**c**). Syn: Synganglion

The first column in Fig. 3 (Fig. 1) should have been labelled ‘Control’ the third column ‘Ivermectin’. The first row should be labelled ‘Intestine’ the second ‘Ventricle’.

The first row in Fig. 4 (Fig. 2) should have been labelled ‘Control’ the second row should be "GP-132 BT", and the third row ‘Ivermectin’. Panel ‘b2’ should have been labelled ‘b1’.

The first column in Fig. 5 (Fig. 3) should have been labelled ‘Control’, the third column ‘Ivermectin’.

The figures are included correctly below and have been updated in the original publication.
